# What drives female objectification? An investigation of appearance-based interpersonal perceptions and the objectification of women

**DOI:** 10.1371/journal.pone.0221388

**Published:** 2019-08-23

**Authors:** Dax J. Kellie, Khandis R. Blake, Robert C. Brooks

**Affiliations:** Evolution & Ecology Research Centre, School of Biological, Earth and Environmental Science, The University of New South Wales, Sydney, Australia; Middlesex University, UNITED KINGDOM

## Abstract

Previous research finds that both men and women perceive sexualized women as lacking in certain human qualities such as mental capacity and moral status. The mechanism underlying this effect, however, is unclear. The present two studies test how appearance-based judgements affect the degree to which a broad sample of women are objectified. In Study 1 (*N* = 279), full-body images of women wearing different clothing outfits were rated by male and female participants on perceived attractiveness, sexual intent and age. In Study 2, male and female participants (*N* = 1,695) viewed these same images from Study 1 and rated them on two dimensions of objectification (agency and patiency). We analyzed associations between these dimensions of objectification and the averaged appearance-based perceptions from Study 1. We find that women perceived as more open to casual sex are attributed less mental capacity and less moral status. We also find that participants tend to associate attractiveness with greater mental and moral status in women, but we find only limited evidence that perceived age influences objectification. Our findings suggest that although positive attractiveness biases may mitigate the amount a woman is objectified, greater female objectification may be prompted by observers’ negative stereotypes of promiscuous women.

## Introduction

The viewing of another person as an instrument to be used for sexual goals is known as objectification [1, 2]. Recent evidence shows that the learned automatic response to objectify women has become culturally ingrained to such a great extent that choosing *not* to objectify women depletes self-regulatory resources and decreases performance in cognitive tasks [3]. In support of this notion, one Australian study on a sample of 81 women found that over one week, each woman reported being targeted for objectification between 3 to 4 times on average and witnessing sexual objectification of other women 9 to 10 times on average [4]. Objectification becomes especially harmful if women internalize these judgements and self-objectify, or consider themselves first as bodies over other personal characteristics [1]. This can lead to negative consequences including heightened body-shame [5] and greater unwillingness to speak in social interactions [6, 7].

Women who are objectified are viewed as less than fully human, perceived to have less of a mind for thoughts or decisions and viewed as less deserving of moral treatment by others [8]. This denial of mental capacity and moral status has been found to have negative repercussions for objectified women, including increasing men’s willingness to commit sexually aggressive actions towards them [9], and decreasing perceived suffering in cases of sexual assault [10–12]. Furthermore, some women are objectified more than others: Women who appear sexualized (e.g., more tightly-fitted, revealing or provocative clothing, greater application of cosmetics), in particular, are objectified more than non-sexualized women [3, 13–15]. Although there is a consensus that sexualized appearance can increase objectification, it is still unclear which judgements based on a woman’s appearance (and in many cases a sexualized appearance) influence the degree to which she is objectified by others. In the current study, we investigate how appearance-based interpersonal perceptions of women affect objectification. Specifically, we test how perceptions of a woman’s sexual intent, attractiveness and age affect attributed amounts of mind and morality.

### Objectification and the attribution of mind and moral status

Viewing another person as an object, or less than fully human, is fundamentally an act of denying that a person has mental abilities and moral status [2, 16]. Previous research shows that when thinking about the mind of another person, people perceive them using two dimensions: *mental agency* and *mental experience* [17]. A person viewed as having mental agency is seen as able to think, plan, or act on their intentions, whereas a person perceived to have mental experience is seen as able to feel or sense emotional and physical pain. Research has also shown people perceive others’ moral status using two similar, connected dimensions: *moral agency* and *moral patiency* [18, 19]. A person seen as having moral agency is viewed as able to commit or be responsible for good or bad deeds, whereas a person perceived to have moral patiency is seen as able to feel or be sensitive to good or bad deeds. However, if a person is perceived to have less of both dimensions of agency and experience, mental and moral, the person’s perceived mental and moral status becomes similar to those of animals, robots or inanimate objects [17, 20]. In other words, a person viewed as lacking in both agency and experience is objectified [10], though the relationship between agency and experience may be more dyadic and complex in nature [21].

Perceiving a person as lacking in mental capacity and moral status can alter the attitudes and behaviors of the perceiver and cause negative consequences for the targeted individual. For example, perceivers are more willing to inflict pain on individuals they perceived to possess less moral patiency [10, 18]. More broadly, the process of denying human qualities of mind and morality is related to many types of prejudices, including racial discrimination [22, 23], reduced empathy for medical patients [24], and negative stereotypes towards people within lower social classes [25]. More specifically for women, evidence shows sexualized women are viewed as lacking in both mental and moral capacity, and as a result, they are seen as less competent [10, 26], less human [26] and perceived to suffer less in sexual assault [11]. Thus, objectification manifested as the denial of mental and moral capacity can negatively affect how targeted individuals, including women, are viewed and treated.

### Interpersonal perceptions and their effects on mind and moral status

Focusing on a woman’s body promotes objectification and decreases perceptions of her mental capacity and moral status [8, 10, 26]. This relationship between body focus and objectification has been demonstrated through multiple lines of research including cognitive [27, 28], visual processing (e.g., inversion effects) [12, 29, 30] and dehumanization research [31]. However, men and women do not objectify women equally, and may not do so for the same reasons [31] or even in the same way [32]. Objectification is regularly discussed as the consequence of sexual goals by men [1, 13] even though objectifying women has been found to be a behavior not exclusively committed by men, but also by women (e.g., [3, 26, 29]). This suggests that although active sexual goals are an important factor explaining many men’s objectification of women [31], there may be alternative factors or more fundamental reasons contributing to greater female objectification by other men and women. Here we discuss three possible reasons as to why men and women might deny other women mental and moral status: because they perceive target women as open to casual sex, because of target women’s perceived attractiveness, and because of target women’s perceived age. All three of these reasons are related to the notion that male sex goal activation and female competition increase objectification, as we explain below.

#### Openness to casual sex

Both men and women objectify women [3, 9, 10, 13–15, 33–35]. One possible explanation as to why women, and primarily sexualized women, are objectified relates to negative attitudes some people hold towards promiscuity. Women who are perceived as more sexually open are found to be more vulnerable to sexual aggression due to lower perceived mental agency [9]. This may not only relate to men viewing sexually unrestricted women as more likely to accept sexual advances [31], but also as a reaction to breaking the gendered social ‘script’ that women engage in less casual sex than men [36, 37]. Evidence also suggests that women who perceive sexualized women as less human view these women as part of a subcategory from which they wish to distance themselves, similar to how an in-group views members of an out-group as less human [31, 38–40]. Women may desire distance from sexualized women not only because they are perceived to perpetuate objectification [31, 39], but because they are assumed to be sexually unrestricted. As a result, a woman’s sexual openness, or more importantly her perceived sexual openness, may cause men and women to make objectifying judgements of her.

Evidence shows that individuals who are interested in a restricted sexual lifestyle may increase their support for institutions and laws which prevent others from behaving promiscuously, as a way to protect their own personal long-term relationships [41–44]. For men, opposition to promiscuity may be associated with a desire to limit their uncertainty about paternity [45, 46]. For women, opposing promiscuity might allow sexually restricted women to increase men’s commitment to long-term marriage, parenthood and resource investment by decreasing the opportunities for men to pursue casual short-term relationships [46, 47]. Given that both men and women frequently interpret revealing attire as a cue of promiscuous behaviour, even though this cue is inaccurate [48], people who oppose promiscuity may objectify sexualized women because they believe sexualized women are more likely to pursue casual sex. Therefore, objectifying judgements of women’s mental capacity and moral status in this instance may materialize due to an aversion towards women who they believe engage in unrestricted sexual behaviour.

#### Attractiveness

Appearance is an influential aspect of many social judgments and biases, including perceived personality traits [49] and professional success [50–52]. Additionally, for women, attractive appearance is important when pursuing romantic partners [53–55]. However, across all societies, men generally are more interested than women in pursuing short-term relationships [56]. Thus, men may be more likely to judge women who are more attractive in relation to their own short-term sexual goals [57]. For example, men but not women who completed a sex goal activation task focused more on attractiveness than competence when asked to choose a partner to complete a mathematical test with [31]. As a consequence, attractive women may be more susceptible to being perceived by men as an object regardless of whether these women are receptive to sexual advances.

However, attractiveness may not only influence perceptions of women due to its relation to activating men’s sex goals; competition between women also tends to focus on comparisons of attractiveness [54, 55, 58]. As a result, a negative bias towards attractive individuals can emerge between same-sex individuals [59]. For example, women and men perceive achievements of attractive same-sex individuals as more due to luck rather than intentions, whereas the same did not hold for the achievements of unattractive individuals [60]. Perceived intentionality is one facet of mental agency [17], suggesting that this negative attractiveness bias may also lead people to view others of the same-sex as having less of a mind if more attractive. Denying mind to other women could also be useful for preventing ego-depletion when comparing oneself to more successful or desirable women [61]. Thus, women may mentally perceive attractive women as more object-like as an intra-competitive response.

#### Age

Evidence suggests that, all else being equal, men prefer younger women, particularly women in their early twenties, as sexual partners [53, 62]. There are many reasons for this finding. From a biological perspective, younger women are more fertile and have more of their reproductive careers ahead of them [63]. Thus, we might find younger women to be objectified more often due to sex goal activation associated with greater fertility. If women are objectified due to perceptions of fertility, we would expect to see women who are both attractive *and* young objectified most by men. From a sociocultural perspective, a woman’s youth may suggest a lack of social power. Powerful individuals perceive subordinates as less human [64, 65], power increases expectations of sexual interest from a subordinate [66], and individuals primed to feel more powerful objectified sexualized women more than low-power individuals [67]. For these reasons, younger women may be more likely to be objectified than older women.

### The current experiment

Despite a large amount of evidence showing that women are objectified, which appearance-based interpersonal judgements lead to greater objectification remains unclear. In the present research we investigate three novel cues that we argue may influence the objectification of women. In Study 1, men and women rated a large, diverse sample of 56 photographs of women on three characteristics: perceived sexual intent, perceived attractiveness, and perceived age. In Study 2, the same photographs of women were rated by a separate group of participants on questions relating to mental and moral agency and mental and moral patiency. Using mixed model regression, we analyze the interpersonal perceptions most associated with objectification. We aim to understand which perceptions of women drive objectification and the degree to which objectification differs between male and female perceivers.

Included as covariates in our analyses are three measures of participant individual difference that may drive objectifying judgements: sociosexuality, mate value, and perceived female economic dependence. Sociosexuality refers to a participant’s attitudes, behaviours, and desires towards casual sex and can influence positive and negative attitudes towards the sexual activity of others [68], thus potentially influencing objectification. Self-rated mate value refers to a participant’s belief that they are an attractive, desirable partner. We included this covariate to test whether women are objectified more often if they are perceived to threaten other women as romantic competitors. Perceived female economic dependence refers to how much a participant believes that women around them depend on men for economic support. Price and colleagues [46] showed that people surrounded by women who depend economically on men hold stronger anti-promiscuity attitudes, demonstrating participants’ perceived socioeconomic environment influences their attitudes towards acceptable behaviours of others. We included this variable to test whether participants who perceive the women around them to depend financially on men may also perceive women to have less mental and moral status, due in part to greater anti-promiscuity attitudes.

Our experiment utilizes a design comparing a large and wide-ranging number of images of women in order to increase the scale, ecological validity, and robustness of our results. Evidence suggests that controlled laboratory experiments often unreliably correlate to real-world effects [69] due to the difficulty of translating between specific paradigmatic settings in controlled scenarios and more variable external settings of the real-world [70]. In the context of this experiment, humans are shown to simultaneously evaluate multiple types of emotional and physical information from faces and bodies together [71–73] and use social comparisons when evaluating themselves and others [74, 75]. This finding suggests that although comparing highly controlled stimuli that differ only in specific targeted traits are essential for understanding fine-scale effects, these may not translate to real-world circumstances. By presenting stimuli that vary on a continuous rather than categorical spectrum we increase the scale of our experiment to ameliorate these issues.

## Methods

This study comprises two experiments performed on the same set of 56 images of women. Ethics for Study 1 and Study 2 was reviewed and approved by the University of New South Wales, Sydney human ethics committee (HREAP 155120) and all participants gave their informed consent to participate in the experiment.

### Data and Supplementary Material

All data, code and Supplementary Material is available and can be accessed on the Open Science Framework (https://osf.io/j3f7m/).

### Target women images

A total of 56 photographs of different women were used as target images (from freedigitalphotos.net and PeopleImages.com). Each picture depicted one full-body image of one woman photographed in front of a white background. All 56 images of target women can be found in the Supplementary Material. Target women ethnicity was 91% Caucasian, 3.5% East Asian, 3.5% African American, and 1.7% South Asian. Images of target women were selected to vary in style of clothing, amount of clothing, and age. Additionally, target women ranged in weight, from thin to overweight. Clothing type varied from casual attire to work attire, and no target women wore religious garments. Clothing ranged from low-coverage (e.g., bikini, skirt, revealing t-shirt) to high-coverage (e.g., long-sleeve buttoned shirt, blazer). Clothing also varied in color and fit. Images of target women ranged in pose, but in all images the face and body were fully visible.

## Study 1

Study 1 investigated differences between target women on perceived likeliness to have casual sex, perceived attractiveness and perceived age. Two hundred and seventy nine participants (146 men, 133 women; *M*_*Age*_ = 36.4, *SD*_*Age*_ = 11.5) were recruited from Amazon’s Mechanical Turk (an online participant recruitment service which allows recruiters to compensate participants for completing experimental research studies) and were paid $1.00 USD to participate. The majority of participants resided in the USA (88.9%) or India (7.89%), and most participants identified their ethnicity as North Western European, British or Irish (34.1%), European Mixed Race (17.6%), or South East Asian (10.4%), with another 10.4% of participants selecting that none of the ethnicities defined their ethnic group. Participants identified as mainly heterosexual (88.5%), with 4.7% identifying as homosexual, 4.7% as between homosexual and heterosexual, 0.7% as asexual, and 1.4% chose not to answer (for full sample demographics, see Supplementary Material).

Each participant was randomly assigned to answer four out of five possible questions about 28 images out of 56 possible images of target women. Participants answered 1 question about 7 target women, displayed individually and successively. Then participants proceeded to answer the next question about another 7 target women, again displayed one at a time, until 4 questions were answered about 28 total target women; 7 answers for each question and 1 rating per target women. Questions and target woman images were randomly selected for each participant. Only 1 target woman image and 1 question were displayed to participants at any time, and no participant rated the same target woman more than once about any question. Although this design was complex, we followed it because we wanted participants to rate a large number of women, but we also aimed to reduce mental fatigue and task disengagement [76, 77]. Additionally, we wished to preserve variation in participants’ responses that can be lost in longer surveys as participants begin to choose identical answers more often in later questions [78, 79]. Preserving this variation in participants’ immediate perceptions towards women was important due to our interest in having participants rate a large number of women and our aim to discerning between dependent variables with substantial theoretical overlap. On average, 27.7 participants rated each target woman on each question (Men: *M* = 14.6, *SD* = 2.73; Women: *M* = 13.3, *SD* = 2.69). See Supplementary Materials for full list of participant numbers that rated each target woman on each question.

Perceived intention to pursue casual sex was measured using a brief three-item version of the Revised Sociosexual Orientation Inventory [48, 80]. The three items were (1) “How likely do you think this person is to have a one-night stand?”, (2) “How likely do you think this person is to have (or have had) a lot of sexual partners?”, and (3) “How likely do you think this person is to require strong relationship commitment before engaging in sexual contact?” (reverse-scored). All items were answered using a 9-point Likert scale (1 = Not at all, 9 = Extremely). Due to our single-item random-allocation study design, participants did not answer all three items about each target woman they rated which prevented us from calculating a measure reliability score between items. However, these items have been repeatedly shown to reliably measure sociosexual orientation [48, 56, 81, 82], so we combined average scores of each question to create one overall rating of perceived sexual intent to pursue casual sex for each target woman (Table 1). The attractiveness of each target woman was assessed using one item: “How attractive do you think this person is?” The item was assessed using a 7-point scale (1 = Not at all; 7 = Extremely). The perceived age of each target woman was measured using a single item: “How old do you think this person is?” The item was assessed using a 9-point scale (1 = 18–24 years, 2 = 25–30 years, 3 = 31–35 years, 4 = 36–40 years, 5 = 41–45 years, 6 = 46–50 years, 7 = 51–60 years, 8 = 61–70 years, 9 = 71 or older). Participant ratings of perceived age and attractiveness were also averaged to create a mean score of perceived age and perceived attractiveness for each target woman (Table 1). Correlations of predictor variables can be found in Table 2.

**Table 1:** Mean ratings of target women for the three predictor variables.

	*M*	*SE*
Perceived Sexual Intent	4.95	.050
Perceived Age	2.81	.038
Perceived Attractiveness	4.59	.037

Note: Perceived sexual intent was measured using a 9-point Likert scale (1 = Low; 9 = High). Perceived age was measured from 1 to 9 (1 = 18–24 years, 2 = 25–30 years, 3 = 31–35 years, 4 = 36–40 years, 5 = 41–45 years, 6 = 46–50 years, 7 = 51–60 years, 8 = 61–70 years, 9 = 71 or older). Perceived attractiveness was measured using a 7-point Likert scale (1 = Low; 7 = High).

**Table 2:** Correlations between three target women predictor variables perceived sexual intent, perceived age and perceived attractiveness.

	Perceived Age	Perceived Attractiveness
Perceived Sexual Intent	-0.58	0.70
Perceived Age		-0.62
Perceived Attractiveness		

Note: Not all participants rated all measures about every target woman, so reported correlations were calculated from average participant ratings of each target woman. Therefore, bivariate correlations, p values and degrees of freedom could not be calculated and are not reported.

We ran analysis of variance (ANOVA) models to test for differences in men’s and women’s ratings of target women on predictor variables. Models included each predictor variable item as the dependent variable, sex of the participant and target woman identity as fixed effects, and participant identity as a random effect. Results indicated that men and women differed significantly in how they rated attractiveness between target women (*F*_55,1302_ = 1.69, *p* = .001, *η*_*p*_^2^ = .05), but overall average attractiveness ratings did not differ significantly. Because participants answered a unique subset of items, we were not able to test differences in ratings between men and women using the combined score of perceived sexual intent items and thus tested each item individually. Female participants rated items one and two of the perceived sexual intent measure lower than men overall (Item 1: *F*_1,1_ = 6.78, *p* = .009, *η*_*p*_^2^ = .009; Item 2: *F*_1,1_ = 17.35, *p* < .001, *η*_*p*_^2^ = .01), but men and women did not differ in which target women they perceived to have more or less sexual intent. Men’s rating of target women as more open to casual sex compared to women’s ratings in two of three sexual intent items may represent a sexual over-perception bias in men compared to women [83]. Ratings of item three of the perceived sexual intent measure and perceived age of target women did not differ between male and female raters.

## Study 2

Study 2 investigated men and women’s mind and moral attribution towards target women. Participants rated the same images of target women as in Study 1 on measures assessing participants’ perceptions of mental and moral capacity.

### Participants

A sample of 1,695 participants (954 men, 741 women; *M*_*Age*_ = 35.4, *SD*_*Age*_ = 11.1) were recruited from Amazon’s Mechanical Turk and each paid $1.00 USD to participate in a survey expected to take 10 minutes to complete. The majority of participants resided in the USA (68.0%) and India (25.4%). Participant ethnicity was primarily North Western European, British or Irish (26.7%), South East Asian (24.7%), or European Mixed Race (13.4%), with another 11.9% of participants electing that none of the ethnicities defined their ethnic group. Participants identified as mainly heterosexual (86.5%), with 6.13% identifying as homosexual, 9.32% as between homosexual and heterosexual, and 1.24% as asexual.

### Mind and moral attribution measures

#### Agency

Agentic perceptions were measured using four items. Two items were selected to assess the mental agency of each target woman [17] and two items were selected to assess the moral agency of each target woman [84]. Mental agency items were chosen by selecting the two items with the highest factor loadings from Gray, Gray and Wegner’s [17] original factor analysis that were also included in the shortened agency scale [9] used by Blake and colleagues to assess mental agency of sexualized vs non-sexualized images of target women. Items were: “How capable do you think this person is at exercising self-restraint over desires, emotions, or impulses?” and “How capable do you think this person is at telling right from wrong?” Moral agency items were chosen by selecting two items from a shortened moral perception scale with the highest factor loadings by Blake and colleagues [9] when used to assess moral perceptions of sexualized vs non-sexualized images of target women. Items were “How much do you believe this person’s achievements and actions are due to their thoughts and intentions, rather than luck and circumstances?” and “In general, how responsible do you think this person is for their actions in life?” All four items were assessed using a 6-point Likert scale (1 = Not at all; 6 = Extremely). Due to the overlap of some items with both mental agency and moral agency concepts, all four items are considered representative of broader perceptions of overall agency and will be collectively referred to as perceptions of *agency*.

#### Patiency

Patiency perceptions were measured using four items. Two items were selected to assess the mental experience of each target woman [17] and two items were selected to assess the moral patiency of each target woman [84]. Mental experience items were chosen by selecting the second and third highest factor-loading items from Gray, Gray and Wegner’s [17] original factor analysis, following Blake and colleagues’ [9] decision to not assess perceptions of “hunger” (the item with the highest factor loading) which we believed was less informative for our study purposes. Items were “How capable do you think this person is at feeling afraid or fearful?” and “How capable do you think this person is at feeling physical or emotional pain?” Moral patiency items, similar to moral agency items, were selected by choosing the two items with the highest factor loadings by Blake and colleagues’ [9] moral perception scale. Items were “How bad do you think you would feel if someone took advantage of this person?” and “How bad do you think you would feel if you manipulated this person?” All four items were assessed using a 6-point Likert scale (1 = Not at all; 6 = Extremely). Due to the overlap of some items with both mental experience and moral patiency concepts, all four items are considered representative of broader perceptions of overall patiency and will be collectively referred to as perceptions of *patiency*.

### Individual differences measures

#### Dependence on income and perceived female economic dependence

Participants who stated that they were in a relationship were asked about how dependent each member in the relationship is on their partner’s income via two items: “How dependent would you say you are on your partner’s financial income?” and “How dependent would you say your partner is on your income”. Participants answered using a 7-point Likert scale (1 = Extremely independent; 7 = Extremely dependent). The Cronbach’s alpha score revealed that this measure was not a consistent trait-level measure of income dependence (Cronbach’s *α* = .24), so we chose to not include this measure in final analyses.

Participants next completed a shortened two-item version of the Perceived Female Economic Dependence Scale [46]. Participants are asked to rate to what extent they believe that the women in their community rely on their male partner for financial income. Participants answered to what extent they agreed on the following statements: “Of the women I know who are in long-term heterosexual relationships, most rely financially on their male partner,” and “Most women I know depend heavily on the money of a male partner, or probably will at some point in their life.” Items were answered using a 7-point Likert scale (1 = Strongly disagree; 7 = Strongly agree). Perceived female economic dependence was calculated by averaging both items to create one overall score (*α* = .88).

#### Sociosexual orientation

Sociosexual orientation was measured using the Revised Sociosexual Orientation Inventory (R-SOI) [80]. Participants answer nine questions to assess their behaviours, attitudes and desires about extramarital and casual sex using a 9-point Likert scale. Examples of items include: “With how many different partners have you had sex within the past 12 months?” (0 to 20 or more), “Sex without love is OK,” (1 = Strongly disagree; 9 = strongly agree), and “How often do you have fantasies about having sex with someone with whom you do not have a committed romantic relationship?” (1 = never; 9 = at least one every day; *α* = .86).

#### Mate value

Self-perceived mate value for each participant was assessed using the Mate Value Scale [85]. The Mate Value Scale is a four-item scale where participants rate themselves on how desirable they believe they are as a partner on a 7-item Likert scale. The four items are: “Overall, how would you rate your level of desirability as a partner on the following scale?” (1 = Extremely undesirable; 7 = Extremely desirable), “Overall, how would members of the opposite sex rate your level of desirability as a partner on the following scale?” (1 = Extremely undesirable; 7 = Extremely desirable), “Overall, how do you believe you compare to other people in desirability as a partner on the following scale?” (1 = Very much lower than average; 7 = Very much higher than average), and “Overall, how good of a catch are you?” (1 = Very bad catch; 7 = Very good catch; *α* = .89).

### Procedure

Participants were first instructed to answer demographic questions and all individual difference measures. Participants were then provided instructions about the structure of the survey and that they would be answering a total of 4 questions about 28 images of target women. Participants also read, “Some of the questions may seem a bit unusual. Please look at each model and try to answer honestly, remembering that this entire survey is anonymous.” The procedure then followed the same design as Study 1 with the only difference being that participants answered four out of eight possible questions about 28 out of 56 possible images of target women. After completing the questionnaire, participants were supplied a debriefing about the nature of the experiment.

Similar to Study 1, we used this design in order to gauge participants’ judgements of a large number of women from a large-scale sample on several measures while minimizing repetition, mental fatigue and exhaustion effects which can reduce valuable variation in participant responses. This approach reduces the risk of exhaustion effects within participants. On average, 106 participants rated each target woman on each question (Men: *M* = 59.6, SD = 5.13; Women: *M* = 46.3, SD = 5.08). See Supplementary Materials for a full list of participant numbers that rated each target woman on each question.

## Results

We conducted eight separate general mixed linear regression models using the lme4 R package [86] (see Table 3 for measure items) to determine whether specific perceived target woman traits explain variation in mind and moral attribution (See Supplementary Material for correlations between dimension items). In order to not overburden participants, and inure them to the questions being asked, each participant answered only a subset of the possible questions about each of the target women that were assigned to them at random. The limitation of this approach is that items cannot be combined to reduce dimensionality, to form overall indices of each construct, or to conduct multivariate tests. As a result, eight different models were necessary. The final eight models included sex (of the participant), perceived intent to pursue casual sex (of the target woman), perceived attractiveness (of the target woman), perceived age (of the target woman) and the interactions between participant sex and each predictor variable from Study 1.

**Table 3:** Means (*M*) and standard errors (*SE*) of participant ratings of target women for agency and patiency items.

Dimension	Item	Question	Men		Women		Overall	
			*M*	*SE*	*M*	*SE*	*M*	*SE*
Agency	1	How capable do you think this person is at exercising self-restraint over desires, emotions, or impulses?	4.04	.03	4.25	.02	4.13	.02
	2	How capable do you think this person is at telling right from wrong?	4.29	.02	4.68	.02	4.46	.02
	3	How much do you believe this person’s achievements and actions are due to their thoughts and intentions, rather than luck and circumstances?	4.16	.02	4.36	.02	4.25	.02
	4	In general, how responsible do you think this person is for their actions in life?	4.36	.02	4.77	.02	4.52	.02
Patiency	1	How capable do you think this person is at feeling afraid or fearful?	4.03	.03	4.50	.03	4.24	.02
	2	How capable do you think this person is at feeling physical or emotional pain?	4.51	.03	5.08	.02	4.77	.02
	3	How bad do you think you would feel if someone took advantage of this person?	4.31	.003	4.70	.03	4.48	.02
	4	How bad do you think you would feel if you manipulated this person?	4.16	.03	4.58	.03	4.34	.02

Note: All items were measured using 6-point Likert scales (1 = Not at all, 6 = Very)

We first ran a Likelihood Ratio Test to determine which predictor variables and interactions best predicted objectification ratings and to avoid overfitting our models (see Table 4). The baseline model included only Target woman and participant identity as random effects. We present each question’s best-fit model according to the Table 4. Participant SOI, perceived female economic dependence and mate value are included in each model as covariates. We found our main significant results remained unchanged when including these covariates in our models (and excluding covariates from our models generally increased effects sizes of significant effects). Therefore, we elected to present models which include covariates because they provide more conservative estimates of effect sizes than models excluding covariates. In all models we found no significant interaction effects between sex of the participant and mental or moral attribution ratings of target women, indicating that there were no significant differences between how male and female participants rated target women.

**Table 4:** Results of Likelihood Ratio Test on models of mental agency, mental experience, moral agency and moral patiency measure ratings of target women.

	Agency												Patiency											
	Self-restraint			Right/Wrong			Responsible			Intentional			Fear			Pain			Take Advantage			Manipulate		
	d.f.	χ_2_	*p*	d.f.	χ_2_	*p*	d.f.	χ_2_	*p*	d.f.	χ_2_	*p*	d.f.	Χ_2_	*p*	d.f.	χ_2_	*p*	d.f.	χ_2_	*p*	d.f.	χ_2_	*p*
Sex	1	12.55	.000	1	39.50	.000	1	9.35	.002	1	42.49	.000	1	26.10	.000	1	49.72	.000	1	23.57	.000	1	22.64	.000
Linear	3	16.62	.000	3	32.54	.000	3	28.35	.000	3	23.95	.000	3	21.09	.000	3	19.79	.000	3	22.42	.000	3	20.31	.000
Sex × Linear	3	16.00	.001	3	20.97	.000	3	17.19	.000	3	9.34	.025	3	5.21	.157	3	1.37	.071	3	8.52	.036	3	4.14	.247

Note: Levels of likelihood analysis:

Sex: Sex

Linear: + Perceived Sexual Intent + Perceived Attractiveness + Perceived Age

Sex × Linear:+ Sex × Perceived Sexual Intent + Sex × Perceived Attractiveness + Sex x Perceived Age

Items were analyzed separately because each participant answered a unique subset of questions about a unique subset of target women, and hence items cannot be combined to form overall indices of each construct.

### Agency

As Table 5 illustrates, the sex of the participant significantly affected 3 out of 4 ratings of target women’s agency, with male participants attributing lower agency than female participants to targets on average. Both male and female participants rated target women perceived as more open to casual sex as less capable of exercising self-restraint, less capable of telling right from wrong, less responsible for their actions in life and less likely to achieve due to intention rather than luck by both male and female participants (*Self-restraint*: *β* = -0.44, *SE* = .17; *Right/Wrong*: *β* = -0.44, *SE* = .13; *Responsible*: *β* = -0.48, *SE* = .15; *Intentional*: *β* = -0.46, *SE* = .15). Both male and female participants were also found to associate target women with greater perceived attractiveness with being more capable of self-restraint, telling right from wrong and being more likely to achieve due to intention rather than luck (*Self-restraint*: *β* = 0.27, *SE* = .09; *Right/Wrong*: *β* = 0.20, *SE* = .07; *Intentional*: *β* = 0.23, *SE* = .08). Additionally, we found male participants viewed target women perceived as more attractive as more capable of self-restraint than female participants (*Self-restraint*_*male*_: *β* = 0.27, *SE* = .09, *F*_1,52.3_ = 10.17, *p* = .002; *Self-restraint*_*female*_: *β* = 0.18, *SE* = .11, *F*_1,51.7_ = 2.91, *p* = .094), more capable of telling right from wrong than female participants (*Right/Wrong*_*male*_: *β* = 0.20, *SE* = .06, *F*_1,52.7_ = 10.47, *p* = .002; *Right/Wrong*_*female*_: *β* = 0.13, *SE* = .08, *F*_1,52.0_ = 2.60, *p* = .113), and more likely to achieve due to intention than female participants (*Intentional*_*male*_: *β* = 0.09, *SE* = .08, *F*_1,51.7_ = 1.31, *p* = .259; *Intentional*_*female*_: *β* = -0.01, *SE* = .09, *F*_1,51.9_ = 0.02, *p* = .894), though these differences were all of marginal significance (Table 5). Target women perceived to be older were perceived as being more capable of telling right from wrong and more likely to achieve due to intention rather than luck than women perceived as younger (*Right/Wrong*: *β* = 0.10, *SE* = .04; *Intentional*: *β* = 0.11, *SE* = .05), but perceptions of target women’s capability of self-restraint and responsibility for their actions in life were unaffected by perceived age (see Table 5). There were no other significant differences between ratings by male and female participants (see Table 5).

**Table 5:** Results of general linear mixed effects model predicting which perceived characteristics of the target women affected objectification ratings using Type III ANOVA.

	Agency												Patiency											
	Self-restraint			Right/Wrong			Responsible			Intentional			Fear			Pain			Take Advantage			Manipulate		
	d.f.	*F*	*p*	d.f.	*F*	*p*	d.f.	*F*	*p*	d.f.	*F*	*p*	d.f.	*F*	*p*	d.f.	*F*	*p*	d.f.	*F*	*p*	d.f	*F*	*p*
Main Effects																									
Sex	1,5429	9.10	.003	1,5422	11.92	.001	1,5462	1.86	.173	1,5430	1.13	.288	1,842	41.82	.000	1,841	60.49	.000	1,5391	4.60	.032	1,842	17.34	.000	
Perceived SI	1,52	9.55	.003	1,52	18.94	.012	1,52	12.01	.001	1,52	10.72	.002	1,53	21.66	.000	1,54	14.47	.000	1,53	24.21	.000	1,53	16.99	.000	
Perceived Attr	1,52	6.14	.017	1,52	6.72	.005	1,52	0.29	.591	1,52	7.71	.008	1,53	1.45	.233	1,54	7.04	.011	1,52	8.01	.007	1,53	1.18	.282	
Perceived Age	1,52	3.76	.058	1,52	7.42	.009	1,52	2.75	.104	1,52	8.98	.004	1,53	3.57	.064	1,53	2.48	.121	1,53	0.01	.920	1,54	0.09	.765	
Sex × p.SI	1,5296	2.27	.132	1,5257	3.21	.073	1,5326	0.05	.821	1,5249	0.05	.822							1,5164	1.16	.279				
Sex × p.Attr	1,5303	2.93	.086	1,5265	1.72	.190	1,5417	3.64	.056	1,5235	0.20	.656							1,5149	0.33	.564				
Sex × p.Age	1,5284	0.28	.599	1,5253	0.13	.715	1,5323	1.19	.276	1,5247	3.10	.079							1,5155	0.43	.510				
Covariates																									
SOI (of Participant)	1,844	3.01	.083	1,842	7.42	.000	1,845	0.99	.321	1,842	0.78	.377	1,842	35.91	.000	1,840	12.77	.000	1,842	1.39	.239	1,842	0.26	.262	
Perceived Fem Econ Dep (of Participant)	1,844	3.80	.051	1,842	16.61	.002	1,847	9.60	.002	1,842	18.21	.000	1,842	7.14	.008	1,840	22.10	.000	1,843	12.64	.000	1,842	19.54	.000	
Mate Value (of Participant)	1,844	16.81	.000	1,842	9.37	.001	1,845	28.57	.000	1,842	2.24	.135	1,842	1.47	.226	1,841	0.69	.407	1,843	1.70	.193	1,842	3.20	.074	

Note: Each model only included variables to the highest level of significance in the Likelihood Ratio Test (Table 4). Items were analyzed separately because each participant answered a unique subset of questions about a unique subset of target women, and hence items cannot be combined to form overall indices of each construct.

### Patiency

As Table 5 shows, the sex of the participant significantly influenced all four items of target women’s mental and moral patiency attribution. Male participants rating target women as less capable of fear or pain than female participants overall and female participants reported feeling worse than male participants in response to the experimental scenario of someone else taking advantage of target women or manipulating the target women (Table 2; Table 5). Target women perceived as open to casual sex were rated by male and female participants as less capable of feeling fear or pain (*Fear*: *β* = -0.33, *SE* = .07; *Pain*: *β* = -0.23, *SE* = .06). In addition, results show that as a target woman’s perceived intent to pursue casual sex increased, male and female participants cared less about someone taking advantage of her or manipulating her themselves (*Take Advantage*: *β* = -0.40, *SE* = .10; *Manipulate*: *β* = -0.30, *SE* = .07). For both male and female participants, a target woman’s attractiveness was positively related to her perceived ability to feel pain but not her ability to feel fear (*Pain*: *β* = 0.09, *SE* = .03). Both male and female participants also cared more about someone taking advantage of a target woman if she was more attractive, but attractiveness did not affect how bad participants felt to manipulate a target woman themselves (*Take Advantage*: *β* = 0.15, *SE* = .05). Overall, perceived age had no significant effects on perceptions of target women’s patiency, though a marginally significant negative effect of perceived age on ability to feel fear was driven by male participants rating older women as less capable of feeling fear compared to younger women (*Pain*_*male*_: *β* = -0.05, *SE* = .02, *F*_1,55.2_ = 5.20, *p* = .026), see Table 5.

### Individual differences covariates

The addition of individual difference covariates did not alter the statistical significance of any of our main results presented in the previous sections. There were several significant covariates, however, and we summarize their overall effects here (for details, see Table 5). Participants who reported greater female economic dependence in their close social contacts were also more likely to rate target women as having lower mind and moral status for seven of eight items. Previous research finds that perceived female economic dependence is associated with anti-promiscuity attitudes [46], and our findings suggest that these perceptions also influence perceptions of target women’s mind and moral status. Participant mate value and SOI positively associated with three of eight mental and moral capacity items, respectively, indicating that people with higher mate value or people who are more sexually open were less likely to objectify women. Participant mate value effects were significant for three of four agency items, whereas two of four patiency items had significant participant SOI effects. This suggests that participants with higher mate value and SOI may make more favourable judgements of target women.

## Discussion

Despite extensive research focusing on sexual objectification, the specific interpersonal judgements that influence how much a woman is objectified remain unclear. In this study we tested whether perceptions of a woman’s openness to sex, attractiveness and age were associated with the degree to which she is perceived to have mental capacity and moral status. We find that the more likely a target woman is judged to pursue casual sex, the less she is perceived to have mental and moral capacity. Attractiveness, by contrast, had inconsistent effects, significantly relating to four of eight total items of mental and moral capacity (two in each overall dimension). However, when attractiveness was found to associate with mental and moral capacity, it was *positively* associated (i.e., more attractive target women were associated with having more mental and moral status). Perceived age significantly related to only one of eight total items, suggesting that youth has limited influence on perceptions of mental and moral capacity of target women. Overall, we find male perceivers attribute less mind and moral capacity to target women compared to female perceivers. Our results also show no significant interaction effects between the sex of the perceiver and mental or moral attribution, indicating that associations men and women make from interpersonal judgements of women are similar. Overall, our results indicate that the degree to which a woman is objectified (a) increases with judgements that a woman has more casual sex, (b) can decrease with perceptions of greater attractiveness, (c) is unrelated to a woman’s perceived age, and (d) is greater by men compared to women overall.

Women judged to be more open to casual sex were associated with being less capable of self-restraint, telling right from wrong, feeling fear, feeling pain, being responsible for their actions, and achieving due to intentions rather than luck. Additionally, the perception that a woman was open to sex was associated with participants feeling better about her being taken advantage of or manipulated in a hypothetical scenario. Our finding supports previous evidence that sexualized appearance can lead to fewer attributed agentic mental states [10] and a loss of perceived humanness [38] by participants. However, our results suggest more specifically that greater objectification of sexualized women may largely, though not entirely, be due to an association between women’s sexualized appearance and assumptions about their sexual behavior.

We also find that both men *and* women attribute women whom they believe are open to casual sex with lower mind and moral capacity. Discerning between these possible motivations for objectifying women is difficult and complex. For men, inferring lower perceived mental and moral capacity in target women may be the result of perceiving these women instrumentally, due to believing these women to be more receptive to sex [31], or negatively, because female promiscuity jeopardizes men’s future paternity certainty [63, 87, 88] and breaks the sexual ‘script’ that women engage in less casual sex than men [36, 37]. For female participants, negative associations between target women’s perceived sexual openness and their mind and moral capacity may constitute a form of disapproval of sexualized women [31, 38, 40], or negativity towards competitors in the dating market [47, 89]. Our experiment provides limited insight into which, if any, of these explanations accounts for why some men and women may associate lower mental capacity and moral status with women perceived as likely to have casual sex. When considering each of these explanations for female objectification, we are reminded that there may not be one overarching reason for objectification and motivations for objectifying women may be multi-layered, depending on each individual’s opinions, gender and cultural norms.

However, our finding that perceptions of lower mental and moral capacity covary with a person’s own belief that the women they know depend economically on men proves instructive. In the U.S.A., both women and men who are surrounded by women who depend economically on men are also likely to hold stronger anti-promiscuity attitudes [46]. Our findings show that men and women who believe women depend economically on men also attribute less mental capacity and moral status to women, suggesting that women may be perceived more negatively overall by individuals who reside in areas where women depend on men, or where women are at least perceived to depend on men. Further experiments are necessary to properly test this relationship, as perceived female economic dependence was used as an exploratory covariate in our analyses. Nonetheless, our findings suggest that men and women hold misogynistic biases towards women if they believe that women around them relay economically on men.

Contrary to our predictions, we find that greater attractiveness is positively associated with perceptions of mind and moral status in women. Both male and female participants associate greater attractiveness with having more self-restraint, achieving due to intention rather than luck, and a greater capacity to feel pain. Furthermore, both male and female participants feel worse about others taking advantage of attractive women compared to unattractive women. Although there is evidence to suggest that more attractive women are more likely to be objectified due to a greater body-focus towards them [57, 84, 90], we find that attractiveness is associated with an increase in many perceived capacities of mind and morality of women. Although we did not predict that attractiveness would positively correlate with perceived mind and morality, men and women have been found to be positively biased towards attractive individuals. The ‘beautiful-is-good effect’, finds that attractive people are assumed to possess more socially desirable personalities [91], and to be more competent than unattractive peers [92]. Maestripieri, Henry and Nickels [51] summarize copious evidence showing positive financial and prosocial biases towards attractive people. Our finding suggests that attractiveness may not be as closely associated with greater female objectification as other perceptions of women such as perceived sexual behaviour. Though surprising, our experimental results replicate similar findings that attractiveness can increase perceptions of agency [21], and we believe this is also likely due to a beautiful-is-good effect on male and female perceivers.

Although evidence suggests that power can activate sexual motivations [66] and increase objectification towards sexualized women [67], our results find that perceiving a woman to be younger does not increase the amount she is objectified. Additionally, our findings suggest perceiving a woman to be younger is not associated with greater objectification due to processes of partner assessment, youthfulness, and fertility. Previous research has found that pre-pubescent girls in sexualized clothing are attributed less mental agency and moral consideration than girls in conservative attire [14, 93], but this may be because they are viewed more like adults when dressed in a sexualized manner [93]. If this speculation is true, perceptions of youth might not stimulate greater power-related objectification due to viewing girls as more subordinate. In accordance, our findings suggest that among adults, a woman’s perceived youthfulness does not alter how much she is objectified. Instead, our finding that older women are associated with greater responsibility for their actions and a greater ability to tell right from wrong suggests mental and moral attribution may be associated with perceptions of life experience.

Overall, we find that compared to women, men attribute less mental and moral agency and patiency to women, on average. This finding may be indicative that men harbor more sexist attitudes than women towards other women. For example, some research shows that stronger sexist attitudes cause men but not women to more easily objectify sexualized women [33]. Additionally, individuals who hold more sexist attitudes are more likely to perceive greater psychological gender differences between men and women [94], possibly reinforcing some men’s perceptions that women are an out-group with lower mental capacity and moral status. Thus, although impressions of a woman’s attractiveness and perceived sexual behaviour may influence both men and women’s perceptions, stronger sexism biases may cause men to hold lower baseline perceptions of women.

### Future directions and limitations

We included perceived female economic dependence as a covariate in our model because of its success in predicting anti-promiscuity views in the U.S.A. [46]. Our finding that perceptions of financial inequality between men and women are associated with female objectification provides a new avenue for investigating the causes and correlates of objectification. Using mind and moral attribution as a proxy measurement of anti-promiscuity could offer new insight into more subtle suppression processes and bring new reasons for suppression to light. Future work examining this predictor may prove insightful.

We attempted to utilize a larger number of stimuli to add more realistic variation to our study. However, the large variation in posture, clothing style, pose and size of women depicted in our images restricts our ability to infer which cues participants relied upon to make judgements. Furthermore, the wide and sometimes uneven variation within our stimuli decreases our ability to make inferences about certain groups. For example, although target women varied in race, only 2 were African American and 3 were South East/East Asian, limiting our ability to make meaningful inferences about minority populations. Additionally, although we make hypotheses about age effects on objectification, a small proportion of our sample was identified to appear older than 35 years of age, due in part to our nonstandard operationalization of age in our age measure. Thus, conclusions about our primarily nonsignificant results regarding age should be taken with consideration to the underrepresentation of older women in our stimuli sample.

The chosen design of our experiment limits our capacity to estimate the correlations among measures at the level of the individual, and thus to draw multivariate insights at the individual difference level. Additionally, due to the online and geographically diffuse nature of our experiment, the associations between attractiveness and judgements of mental or moral capacity that we report may not reflect real-life assessments of women encountered in everyday life, including potential romantic partners or same-sex competitors. Evolutionary and cultural suppression theories rest upon the salience of local mating market conditions, and the heterogenous sample within the current experiment is ill-suited to account for complex cultural differences in objectification behaviours [95]. Thus, at the very least, future studies should address whether our results generalize to the judgments people make of the people they meet in their daily lives, including in exclusively non-WEIRD samples (Western, Educated, Industrialized, Rich, Democratic) [96]. Further, our study exclusively tests the influence of interpersonal judgements on perceptions of women, though some evidence suggests that sexualised men are also targeted by objectifying perceptions [10]. It would be informative if future research asked whether similar relationships also exist for judgments of men, and if not, the ways in which the relationships differ.

## Conclusion

We find that the degree to which a woman is objectified increases with judgements that a she has more casual sex, decreases with perceptions of her greater attractiveness, is unrelated to perceived age, and is greater by male compared to female perceivers. Understanding how multiple judgements by perceivers interact during impression formation of women can help us understand the reasons why sexism and negative female stereotypes remain prevalent within society. Considering the overlap between appearances that are considered attractive or sexualized for women, our results highlight the fine line between positive and negative perceptions that women must constantly consider and balance in their daily appearance choices.

## Supporting information

S1 Table Correlations between items measuring agency and patiency. (DOCX)
