# Supplementary material for: What drives female objectification? An investigation of appearance-based interpersonal perceptions and the objectification of women
Source: PLoS One. 2019 Aug 23;14(8):e0221388. doi: 10.1371/journal.pone.0221388 (PMC6707629; doi:10.1371/journal.pone.0221388)
Supplement: S1 Table — (DOCX) [file pone.0221388.s001.docx]

**Table S1. Correlations between items measuring mental agency, mental experience, moral agency and moral patiency.**

|  |  | Mental  Agency | Mental Experience | | Moral Agency | | Moral Patiency | |
| --- | --- | --- | --- | --- | --- | --- | --- | --- |
|  | **Item** | 2 | 1 | 2 | 1 | 2 | 1 | 2 |
| Mental Agency | 1 | .81 | .11 | .37 | .76 | .83 | .57 | .49 |
|  | 2 |  | .28 | .54 | .89 | .87 | .63 | .59 |
| Mental Experience | 1 |  |  | .40 | .23 | .17 | .42 | .39 |
|  | 2 |  |  |  | .47 | .50 | .47 | .44 |
| Moral Agency | 1 |  |  |  |  | .85 | .63 | .56 |
|  | 2 |  |  |  |  |  | .55 | .48 |
| Moral Patiency | 1 |  |  |  |  |  |  | .47 |
|  | 2 |  |  |  |  |  |  |  |

Note: Mental Agency item 1 = *Self-Restraint*; item 2 = *Right/Wrong*. Mental Experience item 1 = *Fear*; item 2 = *Pain*. Moral Agency item 1 = *Responsible*; item 2 = *Intentional*. Moral Patiency item 1 = *Take Advantage*; item 2 = *Manipulate*. See Table 3 for a complete list of items.
